# Hispanic/Latino Acculturation Profiles and Telomere Length: Latent Class Analysis on a Nationally Representative Sample

**DOI:** 10.3389/fpubh.2021.640226

**Published:** 2021-12-20

**Authors:** Francisco Alejandro Montiel Ishino, Philip McNab, Kevin Villalobos, Jeffrey H. Cohen, Anna M. Nápoles, Faustine Williams

**Affiliations:** ^1^Division of Intramural Research, National Institute on Minority Health and Health Disparities, National Institutes of Health, Bethesda, MD, United States; ^2^Department of Environmental Health & Engineering, Center for a Livable Future, Johns Hopkins Bloomberg School of Public Health, Baltimore, MD, United States; ^3^Department of Anthropology, The Ohio State University, Columbus, OH, United States

**Keywords:** acculturation, telomere length (TL), latent class analysis (LCAs), Hispanic (demographic), complex survey data, Latino (Hispanic)

## Abstract

**Background:** Acculturation profiles and their impact on telomere length among foreign-born Hispanics/Latinos living in the United States (US) are relatively unknown. The limited research available has linked acculturation with shortened telomere length.

**Objectives:** To identify acculturation profiles among a US representative sample of Hispanics/Latinos and to then examine telomere length differences between profiles.

**Methods:** We conducted a latent class analysis among a non-institutionalized US-representative sample of Hispanics/Latinos using the 1999–2002 National Health and Nutrition Examination Survey (*N* = 2,292). The latent variable of acculturation was assessed by length of time in the US and language used as a child, read and spoken, usually spoken at home, used to think, and used with friends (i.e., Spanish and/or English). Telomere length assessed from leukocytes was used as the distal continuous outcome.

**Results:** We identified five profiles: (1) low acculturated [33.2% of sample]; (2) partially integrated [18.6% of sample]; (3) integrated [19.4% of sample]; (4) partially assimilated [15.1% of sample]; and (5) assimilated [13.7% of sample]. Acculturation profiles revealed nuanced differences in conditional probabilities with language use despite the length of time spent in the US. While telomere length did vary, there were no significant differences between profiles.

**Conclusion:** Profiles identified revealed that possible life-course and generational effects may be at play in the partially assimilated and assimilated profiles. Our findings expand public health research using complex survey data to identify and assess the dynamic relationship of acculturation profiles and health biomarkers, while being among the first to examine this context using a person-centered approach.

## Introduction

Acculturation is a dynamic process by which individuals, often immigrants, enter into a new host culture (1, 2) that has both indirect and direct effects on behavior and biology (3, 4). A particular biological marker or biomarker of interest are telomeres—caps of tandem repeat nucleotide sequences at the end of chromosomes that help protect cellular information during replication. The caps diminish as cells divide. As such, telomere length has been used as a biomarker of cellular aging or senescence (5–7). This diminishment has been used to predict accelerated senescence and senescence-associated diseases that increase morbidity and mortality in specific population profiles (8–13), and may be affected by the acculturative process (14).

Senescence-associated diseases include cardiometabolic disorders (e.g., type II diabetes), neurodegeneration (e.g., Alzheimer's and Parkinson's disease), and some types of cancers (15–17). Moreover, the common factors associated with telomere shortening in both animal and human studies are lifestyle and stress. Lifestyle and demographic factors including physical activity, diet, and nicotine use, as well as socioeconomic status have been linked to telomere length (7, 11, 18). A possible mechanistic pathway is that stress causes an oxidative response that affects humans at a cellular level, whereby cells dividing more frequently shorten telomeres and cause apoptosis at higher rates (6, 15). The stressful process of acculturation has been found to have a shortening effect on telomere length (14), and therefore acculturation may be associated with shortened telomeres. Other environmental factors and exposures that have been associated with telomere length may be directly and indirectly related to acculturation; these include biological ancestry categorized by race/ethnicity, poverty, and the built environment of—and environmental exposures from—neighborhoods (19, 20).

Acculturation has been associated with negative health consequences among Hispanics/Latinos (21–24) and other underrepresented groups such as African Americans (25–27). Acculturation may then further exacerbate health disparities in already-vulnerable groups. Among Hispanics/Latinos acculturation has been historically measured by language use. One longstanding validated linguistic acculturation measure is the Short Acculturation Scale for Hispanics (SASH), originally developed by Marin and colleagues (28) and recently validated by Hamilton and colleagues (29). While linguistic acculturation is used as a powerful measure to ascertain an individual's strategy to separate, integrate, marginalize, or assimilate into a new culture in the United States (US) (2, 30), it is unknown how the process is directly related to health biomarkers like telomere length.

Our study builds upon the limited research on acculturation and telomere length, especially among US Hispanics/Latinos. We used latent class analysis (LCA) to, first, identify acculturation profiles among US Hispanics/Latinos based on a nationally representative sample. Our second objective was to examine if differences in telomere length among identified Hispanic/Latino acculturation profiles existed. We proposed that at least three heterogenous profiles of acculturation would be identified. We hypothesized that telomere length would be significantly different between the identified acculturation profiles. We based our hypothesis on a study by Ruiz and colleagues (14) focused on a cohort of Mexican-American pregnant mothers' telomere length as it related to acculturation, discrimination, depression, and their levels of psychosocial stress. Ruiz and colleagues (14) reported a strong relationship between shortened telomere length and mothers' latent variables of negative affect from stressful experiences and acculturation strategies oriented toward US host culture, especially when compared to newly immigrated mothers.

## Materials and Methods

LCA refers to a person-centered technique used to identify unobservable, or latent, profiles within a population (31, 32). We conducted LCA using the National Health and Nutrition Examination Survey (NHANES) US Hispanic/Latino adult (18 years old and older) sample from the 1999–2002 cycles. Hispanic/Latino was operationalized based on NHANES defined race/ethnicity variables that self-identified as Mexican American and Hispanic. The 1999–2002 NHANES cycles allowed us to assess the impact of multiple acculturation factors on mean leukocyte telomere length. The 1999–2002 sample includes useable data from 2,292 Hispanic/Latino participants with acculturation questionnaire data and telomere length data. The Institutional Review Board assessed the research protocol, and as no human participants were involved in this study no approval was necessary. The secondary data analyzed from the 1999–2002 NHANES cycle are publicly available from the Centers for Disease Control and Prevention—National Center for Health Statistics database (https://wwwn.cdc.gov/nchs/nhanes/Default.aspx). A detailed description of the sampling methods and study procedures is available elsewhere (33). Sampling weights were included in all analyses to adjust for survey non-response and sample selection probabilities for the 1999–2002 cycles. Primary sampling units and stratum variables were included to account for the NHANES complex sampling design. All data and analytical files are available upon reasonable request.

### Measures

#### Acculturation

We developed our acculturation latent variable from length of time in the US and language used among Hispanics/Latinos, i.e., Mexican American and other Hispanics. Other Hispanics, as reported in the NHANES, were participants that identified as Hispanic not of Mexican descent. Length of time was split into four categories: (1) <1 year; (2) 1 – <5 years; (3) 5 – <10 years; and (4) 10 years or more (34–36). Language specific questions were modeled after the SASH, which was originally developed by Marin and colleagues (28). The NHANES acculturation questionnaire gauged language used in the context of: (1) as a child; (2) for reading and speaking; (3) at home; (4) to think; and (5) with friends. Response options for all language used questions were: (1) only Spanish; (2) more Spanish than English; (3) both equally; (4) more English than Spanish; and (5) only English.

#### Telomere Length

NHANES collected blood from participants to conduct biomarker and other biological analyses. Telomere data were only publicly available during the 1999–2002 NHANES cycles. Telomere length was assessed from leukocyte assays performed using a quantitative polymerase chain reaction method to measure length relative to standard reference DNA or T/S ratio. See Needham and colleagues (11) for greater detail on lab assay techniques.

#### Sociodemographic and Lifestyle Descriptive Variables

Variables such as gender/sex (i.e., male and female), US citizenship (i.e., no or yes), less than a high school education (i.e., no or yes), smoked at least 100 cigarettes in lifetime (i.e., ever-smoker), and moderate physical activity over the past 30 days (i.e., no or yes) were assessed as descriptives for our sample due to their associations with telomere length (14, 19). Hispanic/Latino composition (i.e., Mexican American or other Hispanic) and citizen of the US (i.e., no or yes) were also assessed.

### Latent Class Analysis

**Figure 1:**
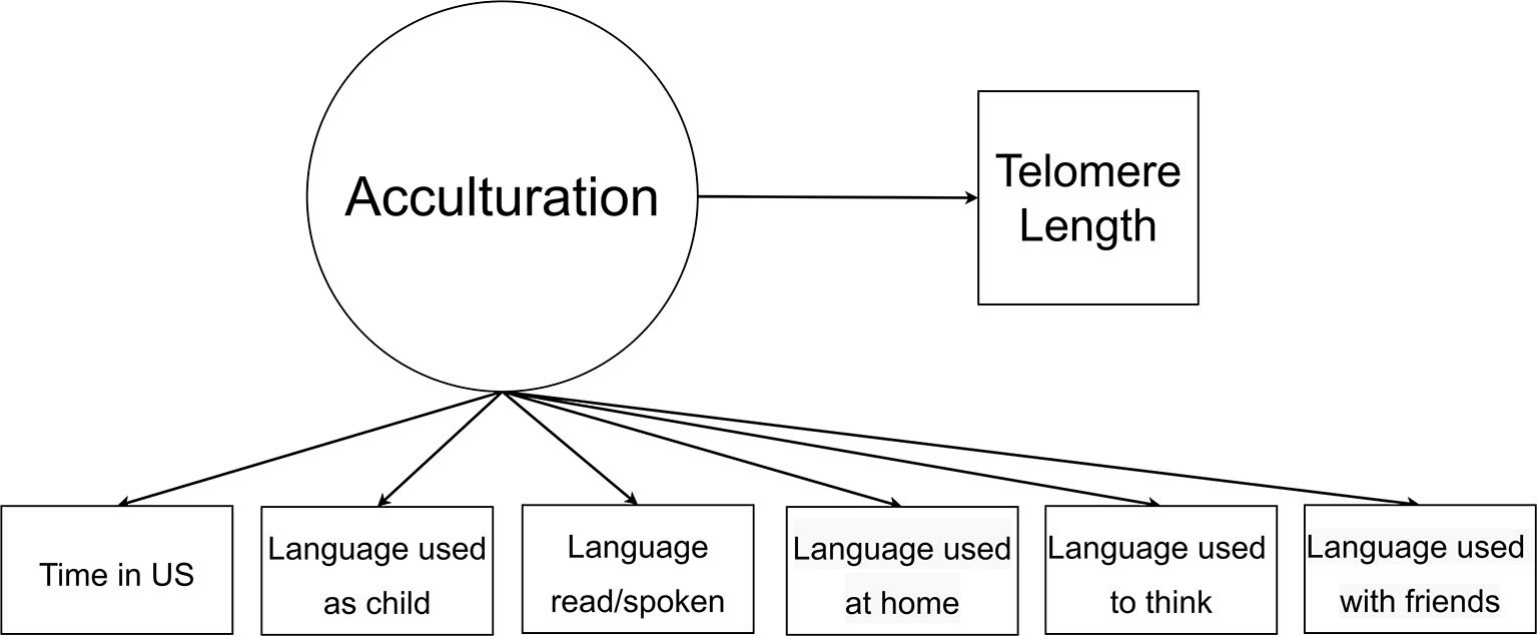
Latent class analysis model of acculturation where the circle represents latent variable and rectangles represent observed indicator variables.

The latent or unobserved variable of acculturation was based on six observed acculturation variables and the distal continuous outcome of telomere length (see Figure 1). We conducted our LCA with Mplus 8.6 (Muthén & Muthén) using a robust maximum likelihood estimator and automatic BCH approach. The automatic BCH method—a modification of the approach developed by Bolck, Croon, and Hagenaars (37)—was used to estimate mean telomere length within each profile and compare differences between profiles.

We used a model comparison approach to determine the number of classes. A one-class model up to a seven-class model was subsequently calculated. The one-class model was calculated to assess fit indices and compare with subsequent models. To assess model fit and reliability, we used Bayesian information criterion (BIC), sample-size-adjusted-BIC (ssaBIC), and entropy (i.e., acceptable quality of classification). We evaluated all models based on fit indices and their practical and theoretical considerations.

## Results

The weighted sample was on average ~40 years of age and equal across gender/sex, with male participants accounting for 49.9 and females 50.1%. The Hispanic/Latino sample was also almost equally weighted between Mexican Americans (50.7%) and other Hispanics (49.3%). Most of the sample were citizens of the US (61.6%) and had more than a High School degree or GED (54.9%), with a mean family poverty income ratio of 2.04. Much of the weighted sample had not smoked more than 100 cigarettes in their lifetime (56.7%), nor engaged in at least moderate physical activity over the past 30 days (64.4%). The Hispanic/Latino mean telomere length was 1.08 T/S ratio. For more details about participant characteristics (see Table 1).

**Table 1:** Descriptive statistics of weighted participant sample (*N* = 2,292).

	**Frequency** **(weighted %)**	**Weighted frequency (SE)**	
**Sex/gender**			
Male	1,092 (49.9)	11,518,928 (1,110,137)	
Female	1,200 (50.1)	11,585,213 (1,273,746)	
**Hispanics/Latinos**			
Mexican Americans	1,875 (50.7)	11,704,451 (1,276,076)	
Other Hispanics	417 (49.3)	11,399,691 (2,134,279)	
**Citizen of the US (*****n*** **=** **2,277)**			
No	906 (38.4)	8,816,781 (1,275,864)	
Yes	1,371 (61.6)	14,164,806 (1,379,033)	
**Less than high school education (*****n*** **=** **2,287)**			
No	933 (54.9)	12,651,015 (1,296,845)	
Yes	1,354 (45.1)	10,373,192 (1,133,168)	
**Ever-smoker (*****n*** **=** **2,284)**			
No	1,302 (56.7)	13,058,383 (1,411,389)	
Yes	982 (43.3)	9,986,520 (1,055,595)	
**Moderate physical activity over past 30 days (*****n*** **=** **2,289)**			
No	1,152 (64.4)	14,876,583 (1,646,121)	
Yes	737 (35.6)	8,207,544 (821,444)	
		**95% CL for Mean**	
	**Mean (SE)**	**Lower**	**Upper**
Age	40.0 (0.592)	38.8	41.2
Family PIR (*n* = 2,060)	2.04 (0.061)	1.92	2.17
Telomere length	1.08 (0.023)	1.03	1.13

*PIR, poverty income ratio; SE, Standard Error*.

Table 2 has the Hispanic/Latino weighted sample acculturation profile. Most participants lived in the US for 10 years or more (60.7%). The largest proportions of the sample used Spanish only as a child (60.2%), to read and speak (29.7%), at home (42.9%), to think (42.0%), and with friends (35.7%).

**Table 2:** Acculturation measure responses of weighted participant sample (*N* = 2,292).

	**Frequency (weighted %)**	**Weighted frequency (SE)**
**Length of time in the US**		
Less than 1 yr in US	72 (8.1)	1,123,041 (284,563)
More than 1 yr, less than 5 yrs	198 (16.4)	2,275,944 (421,384)
More than 5 yrs, less than 10 yrs	194 (14.8)	2,055,003 (266,954)
More than 10 yrs	882 (60.7)	8,409,529 (1,432,145)
**Language used as child**		
Spanish only	1,477 (60.2)	13,649,132 (1,747,330)
More Spanish than English	236 (10.4)	2,364,162 (277,464)
Both equally	207 (10.7)	2,412,563 (293,549)
More English than Spanish	178 (8.1)	1,834,010 (251,671)
English only	175 (10.6)	2,403,690 (263,529)
**Language used to read and speak**		
Spanish only	797 (29.7)	6,732,782 (1,005,467)
More Spanish than English	456 (20.0)	4,529,867 (666,345)
Both equally	413 (20.2)	4,580,644 (449,177)
More English than Spanish	413 (19.9)	4,508,553 (526,949)
English only	196 (10.2)	2,317,946 (296,526)
**Language used at home**		
Spanish only	1,082 (42.9)	9,700,915 (1,588,146)
More Spanish than English	227 (9.7)	2,196,353 (305,020)
Both equally	304 (15.1)	3,419,966 (407,984)
More English than Spanish	271 (12.1)	2,736,351 (335,478)
English only	390 (20.2)	4,582,019 (406,487)
**Language used to think**		
Spanish only	1,046 (42.0)	9,446,896 (1,455,867)
More Spanish than English	215 (8.8)	1,985,948 (23,815)
Both equally	328 (15.8)	3,561,690 (319,875)
More English than Spanish	210 (9.2)	2,057,699 (287,652)
English only	466 (24.2)	5,449,538 (496,264)
**Language used with friends**		
Spanish only	965 (35.7)	8,076,402 (1,230,584)
More Spanish than English	244 (9.7)	2,203,670 (401,562)
Both equally	411 (21.0)	4,762,837 (490,468)
More English than Spanish	248 (12.7)	2,867,081 (343,425)
English only	406 (20.9)	4,726,388 (414,047)

*SE, Standard Error*.

### Latent Class Analysis of Acculturation

The five-class model with low BIC, ssaBIC, and high entropy of 0.891, as well as the most practical and theoretical considerations, was favored (see Table 3).

**Table 3:** Latent class analysis fit criteria of acculturation and telomere length models.

**Model**	**BIC**	**ssaBIC**	**Entropy**
One-class solution	35576.764	35503.688	-
Two-class solution	28410.596	28261.268	0.944
Three-class solution	26075.609	25850.030	0.929
Four-class solution	25309.857	25008.025	0.906
Five-class solution	24869.397	24491.313	0.891
Six-class solution	24801.132	24346.796	0.898
Seven-class solution	24823.808	24293.220	0.897

*BIC, Bayesian information criteria; ssaBIC, sample size adjusted BIC*.

Class 1, or the *low acculturated profile* (33.2% of sample), with mean telomere length of 1.043, was composed of Hispanics/Latinos that had the highest conditional probabilities of being in the US <10 years. Class 1 had the highest conditional probabilities of using Spanish only during childhood (100%), to read and speak (88.0%), at home (99.6%), to think (99.8%), and with friends (96.4%).

Class 2, or the *partially integrated profile* (18.6% of sample), with mean telomere length of 1.102 had a high conditional probability of being in the US more than 10 years (65.5%). Class 2 had the highest conditional probabilities of using more Spanish than English (but not using Spanish exclusively) to read and speak (75.8%), at home (38.3%), to think (34.5%), and with friends (41.5%). The vast majority (86.1%) of this class used Spanish only as a child.

Class 3, or the *integrated profile* (19.4% of sample), with mean telomere length of 1.084 had a high conditional probability of being in the US more than 10 years (86.1%). Class 3 had the highest probabilities of using Spanish and English equally to speak as a child (28.3%), to read and speak (74.6%), at home (58.7%), to think (64.4%), and with friends (74.0%).

Class 4, or the *partially assimilated* (15.2% of sample), with mean telomere length of 1.108 had the highest conditional probability of being in the US more than 10 years (93.2%). Class 4 had the highest conditional probabilities of using more English than Spanish to speak as a child (35.5%), to read and speak (81.1%), at home (49.4%), to think (37.5%), and with friends (47.8%).

Class 5, or the *assimilated* (13.7% of sample), with mean telomere length of 1.101 had the second-highest conditional probability of being in the US more than 10 years (83.6%). Class 5 had the highest conditional probabilities of using only English to speak as a child (68.9%), to read and speak (68.9%), at home (100%), to think (99.9%), and with friends (97.4%). See Table 4 for full detail of the latent class conditional probabilities.

**Table 4:** Conditional probabilities of 5-class solution from latent class model (*N* = 2,282).

	**Class 1:**	**Class 2:**	**Class 3:**	**Class 4:**	**Class 5**
	**Low acculturated**	**Partially integrated**	**Integrated**	**Partially assimilated**	**Assimilated**
	**33.2% (*n* = 757)**	**18.6% (*n* = 424)**	**19.4% (*n* = 442)**	**15.1% (*n* = 346)**	**13.7% (*n* = 312)**
**Length of time in the US**					
Less than 1 yr in US	0.113	0.081	0.000	0.000	0.000
More than 1 yr, less than 5 yrs	0.231	0.112	0.051	0.068	0.164
More than 5 yrs, less than 10 yrs	0.182	0.152	0.088	0.000	0.000
10 yrs or more years	0.473	0.655	0.861	0.932	0.836
**Language used as child**					
Spanish only	1.000	0.861	0.422	0.161	0.017
More Spanish than English	0.000	0.115	0.231	0.196	0.053
Both equally	0.000	0.023	0.283	0.221	0.103
More English than Spanish	0.000	0.000	0.024	0.355	0.138
English only	0.000	0.000	0.040	0.067	0.689
**Language used to read and speak**					
Spanish only	0.880	0.038	0.006	0.000	0.000
More Spanish than English	0.120	0.758	0.053	0.019	0.000
Both equally	0.000	0.195	0.746	0.133	0.037
More English than Spanish	0.000	0.010	0.149	0.811	0.275
English only	0.000	0.000	0.046	0.037	0.689
**Language used at home**					
Spanish only	0.996	0.416	0.065	0.050	0.000
More Spanish than English	0.003	0.383	0.096	0.023	0.000
Both equally	0.001	0.082	0.587	0.160	0.000
More English than Spanish	0.000	0.028	0.184	0.494	0.000
English only	0.000	0.091	0.068	0.273	1.000
**Language used to think**					
Spanish only	0.988	0.425	0.054	0.006	0.000
More Spanish than English	0.007	0.345	0.074	0.032	0.000
Both equally	0.005	0.185	0.644	0.028	0.000
More English than Spanish	0.000	0.041	0.116	0.375	0.001
English only	0.000	0.004	0.112	0.559	0.999
**Language used with friends**					
Spanish only	0.964	0.189	0.018	0.000	0.000
More Spanish than English	0.022	0.415	0.029	0.021	0.003
Both equally	0.011	0.260	0.740	0.122	0.003
More English than Spanish	0.003	0.088	0.145	0.478	0.020
English only	0.000	0.047	0.068	0.380	0.974
**Telomere length**					
Mean T/S ratio (SE)	1.043 (.033)	1.102 (.037)	1.084 (.034)	1.108 (.033)	1.101 (.031)

*SE, Standard Error*.

#### Mean Telomere Length Across Latent Classes

Telomere length by profiles were compared. The automatic BCH approach revealed that the equality test of means across classes for the overall differences was not significant (*x*^2^ = 4.54, *df* = 4, *p* = 0.34). See Appendix for Supplementary Table 1 for between class mean comparisons.

## Discussion

Our study using a nationally representative sample identified five profiles of Hispanic/Latino linguistic acculturation and their respective telomere length. In using the five SASH items in conjunction with time spent in the US we were able to create more dynamic profiles based on the acculturative process. The acculturative process involves language and behavioral norm acquisition from prolonged contact with the host culture (2, 30). US Hispanics/Latinos have been reported to have various acculturation strategies that include marginalization (i.e., rejection of both native and host culture), segregation (i.e., non-integration into the host culture), enculturation, integration, or assimilation (24, 38–40). Enculturation is often operationalized as reintegration or relearning of an individual's native culture (24). Integration is the process where an individual adopts aspects of the host culture without the loss of their native culture (1). Assimilation is the process where the individual replaces aspects of the native culture with those adopted from the host culture (1). The process of assimilation in the acculturative process can be a source of high psychosocial stress due to feelings of otherness and discrimination (34, 40, 41), as well as the lifestyles changes that lead to a loss of social support and unhealthy behaviors (23, 42, 43). The psychosocial effects are prominent in subsequent generations, as protective health behaviors and support structures from the native culture diminish. Moreover, Hispanic/Latino groups are reported to experience worse health outcomes as they become more similar to their US counterparts (24).

Language serves as a primary factor to integration into a new host culture (28). For instance, in reviewing the low acculturated (Class 1) profile of US Hispanics/Latinos that almost exclusively used Spanish regardless of time spent living in the US, two patterns emerged in this profile that will require further examination. First, the low acculturated profile had an approximate conditional probability of 34% to be in the US <1 year and between 1 and <5 years, which would explain the Spanish only linguistic acculturation. Newly arrived immigrant groups will learn the host country's culture and language, or that is the expectation of the host country for the newly immigrated (1, 2, 44). Second, the highest conditional probability of time spent in the US, was 47% on 10 years or more. The high conditional probability of being in the US for a decade or longer in juxtaposition of Spanish only linguistic acculturation may be indicative of low acculturation due to marginalized or segregated individuals as described by Berry (1, 44). The low acculturated profile represented the largest subgroup across all identified profiles.

While it is difficult to ascertain whether persons are marginalized and segregated in our study, it should be noted that low acculturated individuals have been reported to experience worse mental health outcomes (38). The effect of low acculturation and telomere length is less clear. Some studies have found linear associations with lower acculturated groups and decreased telomere length when compared to more acculturated or assimilated groups (45, 46). Specifically, a study by Ruiz and colleagues (14) reported that among pregnant Mexican mothers there is a complex association between low acculturation and decreased telomere length in the presence of psychosocial stress. Inversely, a study among Mexican women in the US reported that among the high acculturation group, longer telomeres were associated with increased percentage body fat but reported no association with low acculturated women (47). In our study, while we found that the low acculturated group had the lowest mean telomere length, it was not significantly different from all other acculturation profiles. Future studies must consider other factors from joint pathologies or other disease etiologies, in addition to environments that may facilitate acculturation strategies such as marginalization or segregation.

Nevertheless, environmental data from built-environmental features, community compositions, and neighborhoods were not available for use in our model, but we must acknowledge their role in linguistic acculturation. Discrimination and discriminatory policies that may be linguistically biased must also be considered to understand low acculturation in context of segregation and marginalization in an environmental context (1, 2, 30). The neighborhood environment, however, may have protective or detrimental effects to the newly introduced immigrant's health based on individual and family language usage (48–50). Neighborhood racial and ethnic composition may also have a role in linguistic acculturation, and their selected acculturation strategy (51, 52).

By contrast, the partially integrated (Class 2) spoke more Spanish than English and were more likely than the low acculturated class to have lived in the US 10 or more years (65.5% conditional probability). The integrated (Class 3) was comprised of Hispanics/Latinos living in the US 10 or more years that used English and Spanish equally, at home, to think, to read and speak, and with friends. The integrated profiles may indicate biculturalism or adaptive profiles. While the integrated profiles are often adaptive, there are some mental and physiological health concerns. Hispanics/Latinos in this adaptive bicultural process adopt customs and language norms that will benefit their integration into the larger US culture (24, 38, 40). Issues concerning identity are at the crux of this process as the degree to which Hispanics/Latinos have a choice to enculturate, integrate, or assimilate is unknown as are the health consequences.

Most individuals (57.8%) in the integrated class used at least some English during childhood—although a plurality (42.2%) of them solely spoke Spanish during this period of their lives. More than two-fifths (42.2%) of people in this class used Spanish only as a child. The integrated class indicated possible language use change over time. Interestingly, even though the conditional probability of using more English than Spanish to think was highest for this class, a majority of people (55.9%) in the partially assimilated (Class 4) reported using English only to think. The partially assimilated (Class 4) were those with the highest likelihood of living in the US 10 or more years and using more English than Spanish but not exclusively English to read and speak, at home, and with friends. The partially assimilated class illustrated the importance of social context for language use, as many individuals continued to use Spanish frequently in their lives despite reporting English use for thinking. The assimilated (Class 5) were those living in the US 10 or more years and had the highest conditional probability to speak only English as a child. The majority of the assimilated exclusively spoke English only as a child, to read and speak, at home, to think, and with friends.

Assimilation in first generation individuals can be stressful, compared to subsequent generations (24, 53). The process of assimilation in subsequent generations is often classified as either congruent or dissonant between parents and children (54). The assimilated classes from our findings could be indicative of second or subsequent generations of Hispanics/Latinos in our sample. Findings suggest a generational effect in the partial assimilation (Class 4) and assimilation (Class 5) as the profiles have an overall lower probability of using Spanish and a higher probability of speaking more English as children, respectively, to other profiles. The partially assimilated primarily had a higher probability of speaking more English than Spanish as children (i.e., 35.5% conditional probability). To contrast, the assimilated profile had the highest probability to speak English only as children (68.9% conditional probability).

Overall, using our LCA on a US representative sample of Hispanic/Latinos we identified heterogenous classes of acculturation that may reveal differences in experiences and processes, which were theoretically suggested in the literature. Linguistic acculturation can be a powerful indicator of risk, but more may be needed to detect telomere length differences between subgroups. While various reasons can be attributed as to why significant telomere differences were not detected, linguistic acculturation may be indicative of other psychosocial stressors and adaptive strategies. For instance, while language can be a source of insecurities and discrimination it can also be key to facilitate equitable access to healthcare and mental health services. In context of our findings, the largest subgroup was low acculturated with almost exclusive Spanish use while having lived in the US for 5 or more years. This may be indicative that a large proportion of US Hispanics/Latinos speak only Spanish, which may affect their access and quality of health services. Nonetheless, linguistic interventions will not be enough to mitigate the possible disparity. Spanish interpreters or translated health materials are a start but literacy and cultural empathy are critical to intervene or prevent excess risk by incorporating customs, norms, and behaviors that are conducive to health among US Hispanics/Latinos. To mitigate disparities and promote health equity, future studies must collect environmental and community data, as well as biomarkers of risk to create more comprehensive models. These comprehensive models may be used to confirm if profiles from our findings remain consistent or detect telomeric differences. Telomere differences may be indicative of increased morbidity and mortality among certain subpopulations (55). As such, the importance of detecting telomere length by acculturation may, for instance, be the critical difference in identifying cancer risk or preventing cancer among Mexican Americans (56). Lastly, in using more comprehensive person-level approaches we can model risk contextually to not only understand syndemic vulnerabilities—the synergy of disease outcomes interacting with comorbid conditions, as well as other social, cultural, biological, and environmental determinants in context of human rights (57)—but to also help develop tailored interventions and prevention programs for those at increased risk from among the most at-risk (24, 47, 53, 54).

## Limitations and Strengths

Our study had four major limitations. The first was that the data, although nationally representative, were cross-sectional; therefore, we were unable to examine changes within the sample over time. Still, various published studies and reports have demonstrated that the data are of acceptable quality (11–13, 58). Second, acculturation is a complex, dynamic multidimensional process. Acculturation measures that exclusively use linguistic measures to assess the process have been critiqued (30, 53). Nonetheless, the validated SASH measure serves as a measure to assess a facet of acculturative strategies (29). Third, the telomere data were not from the current NHANES as they are only accessible from the 1999–2002 cycles. The fourth limitation concerns the meaningfulness of telomere length data in health outcomes research. While telomere research continues to be refined, the interpretation of telomere length and outcomes on morbidity and mortality are less direct (11–13, 59) and may explain why no significant telomere length differences were detected. Although more advanced methods from a manual BCH auxiliary regression approach in Mplus (37) would allow us to test the effects of covariates on classes, we would not be able to test differences of telomere length by class. Additionally, the manual approach would not permit us to test telomere length differences between profiles.

Our study also possessed notable strengths, as it is among the first to focus on acculturation and telomere length, a complex biomarker of senescence and risk. We used the most current techniques for available software packages to assess acculturation profiles and biological processes using complex survey design data of US Hispanics/Latinos. Future research will incorporate multiple acculturation factors beyond language measures to assess its impact on health biomarkers in the context of syndemic vulnerability and risk. A syndemic and latent variable approach will be critical as various factors co-occur and synergize to affect biological processes occurring in tandem without a priori categorization to capture person-centered contexts.

## Conclusion

We identified five Hispanic/Latino acculturation profiles at possible differential risk of shorter telomere length, but no significant telomere length differences emerged. Specifically, our findings will contribute to the emerging literature on the relationship between acculturation profiles and associated biomarkers of health and disease. Our findings and approach provide a way to identify groups most at-risk in already vulnerable subpopulations. Through this work we can understand contextual risk, as well as develop prevention programs and targeted health interventions among US Hispanic/Latino groups. The implications of this research will be to examine the dynamic effects of acculturation using comprehensive models of risk biomarkers to develop prevention programs in order to mitigate health disparities and move toward health equity.

## Data Availability Statement

Publicly available datasets were analyzed in this study. This data can be found at: https://wwwn.cdc.gov/nchs/nhanes/Default.aspx.

## Author Contributions

FM: conceptualization, data curation, methodology, formal analysis, visualization, roles, writing—original draft, and writing—review and editing. PM: roles, writing—original draft, conceptualization, and writing—review and editing. KV: conceptualization and writing—review and editing. JC: resources, roles, writing—original draft, supervision, and validation. AN: roles, writing—original draft, writing—review and editing, supervision, and validation. FW: project administration, resources, software, supervision, writing—review and editing, and validation. All authors contributed to the article and approved the submitted version.

## Funding

The efforts of KV, FM, AN, and FW were supported by the Division of Intramural Research - National Institute on Minority Health and Health Disparities, National Institutes of Health.

## Author Disclaimer

The content is solely the responsibility of the authors and does not necessarily reflect the views of the National Institutes of Health.

## Conflict of Interest

The authors declare that the research was conducted in the absence of any commercial or financial relationships that could be construed as a potential conflict of interest.

## Publisher's Note

All claims expressed in this article are solely those of the authors and do not necessarily represent those of their affiliated organizations, or those of the publisher, the editors and the reviewers. Any product that may be evaluated in this article, or claim that may be made by its manufacturer, is not guaranteed or endorsed by the publisher.
