# Supplementary material for: Hispanic/Latino Acculturation Profiles and Telomere Length: Latent Class Analysis on a Nationally Representative Sample
Source: Front Public Health. 2021 Dec 20;9:640226. doi: 10.3389/fpubh.2021.640226 (PMC8722469; doi:10.3389/fpubh.2021.640226)
Supplement: Supplementary file 1 [file Table_1.docx]

Appendix

Supplementary Table 1

| Supplementary Table 1. Equality tests of means across classes using the BCH procedure with 4 degrees of freedom for the overall test | | |
| --- | --- | --- |
|  | x2 | *p*-value |
| Overall test | 4.54 | 0.34 |
| Class 1 vs. 2 | 2.64 | 0.10 |
| Class 1 vs. 3 | 1.86 | 0.17 |
| Class 1 vs. 4 | 3.23 | 0.07 |
| Class 1 vs. 5 | 1.90 | 0.17 |
| Class 2 vs. 3 | 0.17 | 0.68 |
| Class 2 vs. 4 | 0.02 | 0.88 |
| Class 2 vs. 5 | 0.00 | 0.98 |
| Class 3 vs. 4 | 0.48 | 0.49 |
| Class 3 vs. 5 | 0.15 | 0.70 |
| Class 4 vs. 5 | 0.03 | 0.87 |
|  |  |  |
